# JG6, a novel marine-derived oligosaccharide, suppresses breast cancer metastasis via binding to cofilin

**DOI:** 10.18632/oncotarget.1959

**Published:** 2014-05-12

**Authors:** Xun Huang, Danni Sun, Qiuming Pan, Weiwei Wen, Yi Chen, Xianliang Xin, Min Huang, Jian Ding, Meiyu Geng

**Affiliations:** ^1^ Division of Anti-Tumor Pharmacology, State Key Laboratory of Drug Research, Shanghai Institute of Materia Medica, Chinese Academy of Sciences, Shanghai, P.R.China

**Keywords:** JG6, cofilin, actin, migration, metastasis

## Abstract

Cofilin, an actin-binding protein which disassembles actin filaments, plays an important role in invasion and metastasis. Here, we discover that JG6, an oligomannurarate sulfate, binds to cofilin, suppresses the migration of human breast cancer cells and cancer metastasis in breast cancer xenograft model. Mechanistically, JG6 occupies actin-binding sites of cofilin, thereby disrupting cofilin modulated actin turnover. Our results highlight the significance of cofilin in cancer and suggest JG6, a cofilin inhibitor, to treat metastatic cancer.

## INTRODUCTION

Cancer metastasis constitutes the major cause of death in cancer patients. The cellular basis of cancer invasion and metastasis is the up-regulated cell motility of cancer cells, which is the hallmark of invasion and an essential step in metastasis[1]. Targeting tumor cell migration or invasion is a potential strategy for anti- metastasis[2, 3]. The multi-step cell migration of invasive and metastatic cancer cells is initiated by the formation of membrane protrusions in response to migratory and chemotactic stimuli. The driving force for such membrane protrusion is localized polymerization of submembrane actin filaments[4, 5].

Actin framework is widely accepted as the basic engine for cell motility. The molecular machinery controlling the assembly/disassembly of actin filaments consists of several actin-binding proteins that regulate the dynamic behavior of actin cytoskeleton. Of them, the ubiquitously distributed cofilin protein is the most important regulator for accelerating actin-filament turnover and generating free barbed ends by facilitating pointed end depolymerization and filament severing[6]. In migrating or invading cells, cofilin is localized to the protrusion of the cell membrane such as lamellipodia, invadopodia and filopodia that initiates cell movements and determines cell polarity[7, 8]. This relocalization is critical for the regulation of cell motility and cellular processes such as chemotaxis, endocytosis, and cell division, which are important for both cell physiology and cancer development.

Although there is as yet no direct evidence for a role of deregulated cofilin activity in the etiology of human cancers, the increased activity of the cofilin pathway and its output has been demonstrated in cancer cells[9]. There are at least three stages of cancer progression in which cofilin and its regulation are likely to be important: the initial process of cell transformation[10], increased cell motility during metastasis[11, 12], and cell division[13]. Results to date also indicate that cofilin might be closely involved in cancer development[14, 15]. These together suggest the therapeutic opportunities of targeting cofilin in cancer therapy. In a cofilin-orientated anti-cancer strategy, it is important to note that functional cofilin located at the dynamic protrusion is only a very small proportion of total cofilin, and several tightly uncoupled mechanisms are involved in regulating cofilin activity, which may pose challenges for its effective intervention in cancer therapy[15].

**Fig1:**
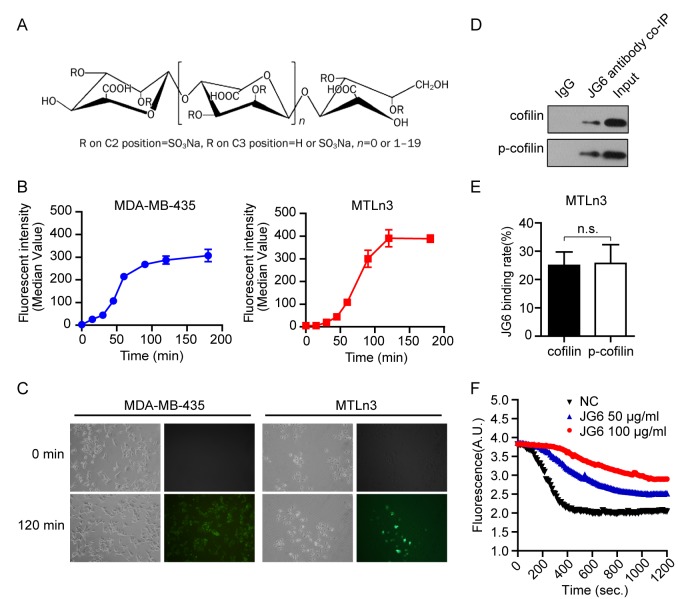
JG6 binds to cofilin and inhibits its actin-severing activity (A) The chemical structure of JG6. (B) Cancer cells were seeded and 100 μg/ml JG6-FITC was added. After incubated with JG6-FITC for the indicated time, cells were harvested and washed three times with PBS, and analyzed by FCM. Data were analyzed with CellQuest software and shown as means±S.E. of three independent experiments. (C) Cancer cells were seeded and 100 μg/ml JG6-FITC was added. Before and after the addition of JG6-FITC, cells were analyzed and photographed under a fluorescence microscope. Five parallel samples were prepared in each group and results are representative of three separate experiments. (D) Binding of JG6 and cofilin or pSer3-cofilin were co-immunoprecipitated with JG6 antibody, followed by immunoblotting using cofilin or pSer3-cofilin antibody. IgG co-immunoprecipitation is used as a negative control. (E) The JG6 binding rate was calculated by measuring the intensity of co-IP bands normalized with the intensity of input bands, which was quantified by ImageJ software. Data are means±S.E. of three independent experiments. *n.s.*, not significant. (F) 4 μM pyrene-actin was polymerized for 12 h at 20 °C. Depolymerization was initiated by a 12-fold dilution with 2 μM cofilin and 50 or 100 μg/ml JG6. The decline in fluorescence was monitored immediately.

JG6 (Fig. 1A), a novel marine-derived oligosaccharide previously discovered binding to extracellular factors and inhibit chemotaxis[16], was noted in an affinity chromatography analysis that indicated its binding affinity to cofilin. The aims of the present study were to further validate the intracellular targets of JG6 and assess the potential anti-migration and anti-metastasis activities of JG6. Our studies have demonstrated that JG6 suppresses the depolymerization/severing activities of cofilin on F-actin via occupying the actin-binding sites of cofilin, which largely accounts for the suppression of breast cancer migration by JG6. Our results promise JG6 in particular and oligosaccharide possibly in general, to be a new and hitherto unrecognized therapeutic class in cancer therapy, and further support cofilin as an emerging target in cancer therapy.

## RESULTS

### JG6 binds to cofilin and inhibits its actin-severing activity

Our pilot experiment using JG6 affinity chromatography in combined with liquid chromatography-tandem mass spectrometry (LC-MS/MS) analysis has suggested that JG6, a novel marine-derived oligosaccharide possessing interesting bioactivities, binds to actin-regulating protein cofilin (Table. S1). Before proceeding to confirming the intracellular binding of JG6 to cofilin, we first tested whether JG6 was able to enter the cells. JG6 was visualized by conjugated to fluorescein isothiocyanate (FITC). Flow cytometry analysis showed that fluorescent intensity of both MDA-MB-435 and MTLn3 cells treated with JG6-FITC was increased (Fig. 1B), indicating a significant amount of JG6 binding to or enter these cells. Further experiment using fluorescence microscopy analysis indicated the entry of JG6 into these cancer cells (Fig. 1C).

We then took the advantage of a specific antibody raised against JG6 to verify its binding to cofilin in cancer cells. Immunoprecipitaion was performed using JG6 antibody in cellular extracts derived from breast adenocarcinoma MTLn3 cells, which were pretreated with 100 μg/ml JG6 for 24 hours. The presence of cofilin in the immunoprecipitates was examined using immunoblotting. Cofilin was clearly detected in the complexes pulled down by JG6 antibody. Moreover, it appeared that JG6 bound equally to un-phosphorylated and phosphorylated forms of cofilin (Fig. 1D and 1E), suggesting that phosphorylation signaling is not involved in JG6 binding. We then were intrigued to investigate the biological significance behind.

Cofilin is known to importantly regulate the dynamics of the actin cytoskeleton via its actin-severing and depolymerization activity[17, 18]. We firstly examined whether JG6 treatment affected the severing and depolymerization activity of cofilin. Polymerized actin was incubated with purified cofilin to initiate depolymerization, which was detected by the decline in fluorescence. JG6 treatment markedly suppressed the depolymerizing/severing activities of cofilin, as shown by a reduced decrease in fluorescence intensity compared with the untreated group (P < 0.01) (Fig. 1F). This finding suggested that binding of JG6 to cofilin impaired the biological function of cofilin.

### JG6 disrupts cofilin-actin interaction

**Fig2:**
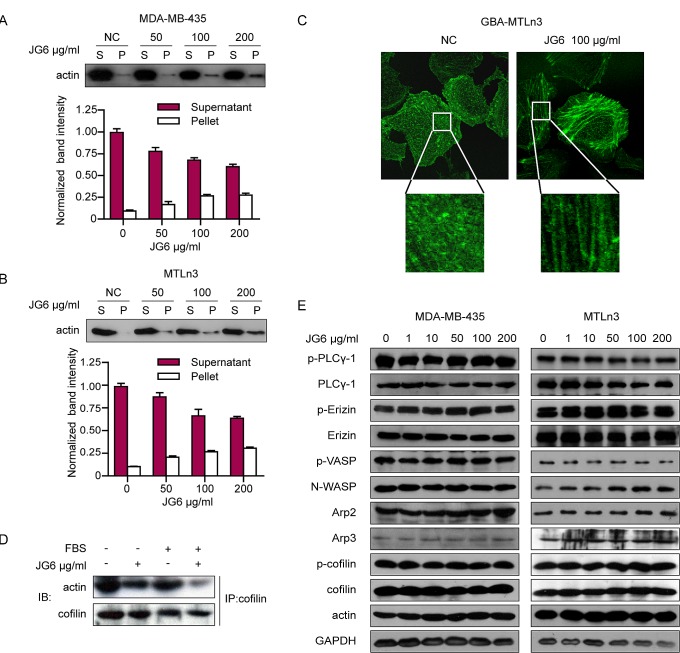
JG6 suppresses the depolymerization/severing activities of cofilin on F-actin (A&B) MDA-MB-435 and MTLn3 were incubated with JG6 (0, 50, 100 and 200 μg/ml) for 24 hours. Cell extracts were extracted for F-actin (F)/G-actin (G) fractionation assay as described [11]. The supernatant and pellet were separated, followed by immunoblotting using anti-actin antibody. Band intensity was quantified by ImageJ software. Data are shown as means±S.E. of three independent experiments. (C) GBA-MTLn3 cells were incubated with JG6 100 μg/ml for 24 hours and live cell images were photographed under a confocal microscope. (D) MTLn3 cells were incubated with JG6 100 μg/ml for 24 hours and then stimulated with 10% FBS or not, binding assay of actin and cofilin was analyzed by immunoprecipitation using anti-cofilin antibody and then subjected to immunoblotting analysis for actin. (E) MDA-MB-435 and MTLn3 cells were pretreated with the indicated concentration of JG6 for 24 hours and lysated for immunoblotting analysis with indicated antibodies.

The reduction in the rate of actin depolymerization suggests a blockage in reorganizing F-actin into monomeric G-actin molecules. We hence assessed the effect of JG6 on the ratio of G-actin to F-actin[19-22]. MDA-MB-435 and MTLn3 breast cancer cells were fractionated into supernatant and pellets to measure the proportion of F-actin to G-actin. As shown in Fig. 2A and 2B, JG6 treatment increased F-/G-actin ratios in both MDA-MB-435 and MTLn3 cells compared to those of untreated control cells. The increase in F-/G-actin ratio was attributed to the increase in F-actin whereas decrease in G-actin levels.

Defect in actin depolymerization may also result in the accumulation of actin filament in the form of stress fibers in cancer cells[23]. We thus examined the impact of JG6 on stress fibers. The stress fibers in the breast cancer cells were visualized by conjugating actin to green fluorescent protein (GFP). Live cell imaging in GBA-MTLn3 cells expressing GFP-actin showed that few actin stress fibers were observed in the control groups whereas fan-shaped stress fibers were scattered all around GBA-MTLn3 cells after exposure to JG6 (Fig. 2C), suggesting that JG6 treatment significantly increased the formation of cellular stress fibers.

Our results thus far have suggested that JG6 binds to cofilin and inhibits its actin-severing and depolymerization activity. It has been known that F-actin depolymerizing/severing activities of cofilin are regulated at the binding sites. We thus presumed that JG6 might compete with actin in accessing to binding sites of cofilin. To test this possibility, we measured the effect of JG6 on the interaction between cofilin and actin. MTLn3 cells were incubated with JG6 100 μg/ml for 24 hours. The detection of actin-cofilin interaction was facilitated by serum stimulation, which was known to accelerate the dynamics of the actin cytoskeleton and in turn increase its interaction with cofilin[9]. Cell lytase was analyzed by immunoprecipitation using anti-cofilin antibody and then subjected to immunoblotting analysis for actin. The co-immunoprecipitation assay revealed that JG6 treatment disrupted actin-cofilin interaction in the presence of serum stimulation (Fig. 2D).

In addition to cofilin, the assembly and disassembly of actin filaments are regulated by a variety of actin-binding proteins. We also investigated the impact of JG6 on these proteins. As shown in Fig. 2E, JG6 treatment barely affected other actin binding proteins or their phosphorylation in both MDA-MB-435 and MTLn3 cells.

It suggests to us that JG6 can disrupt the interaction between cofilin and actin, and thereby inhibit actin turnover by restricting actin depolymerization.

### The binding mode of JG6 to cofilin

**Fig3:**
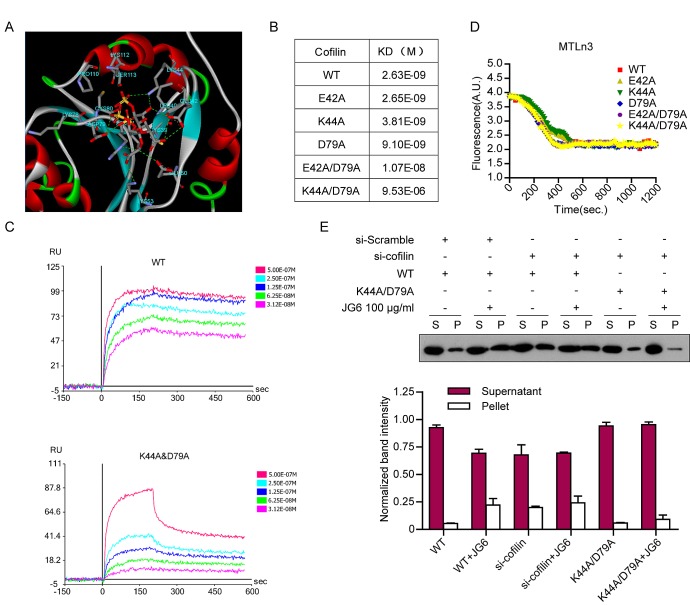
The binding mode of JG6 to cofilin (A) The binding pattern between JG6 with cofilin. Computer molecular simulation was applied with a JG6 trimer. The colored stick structure reflects the oligosaccharide and the colored line and band around it indicate the conformation and secondary structure of cofilin. The potential interaction AA sites on cofilin were marked. (B) Binding between JG6 and cofilin WT or mutants. (C) The binding curves of JG6 with cofilin WT and K44A/D79A were determined using surface plasmon resonance. (D) 4 μM pyrene-actin was polymerized for 12 h at 20 °C. Depolymerization was initiated by a 12-fold dilution with 2 μM WT or mutant cofilin. (E) MTLn3 cells transfected with cofilin siRNA followed with cofilin WT or mutation plasmids were incubated with JG6 100 μg/ml for 24 hours. F-actin (F)/G-actin (G) fractionation and immunoblotting analysis were similar to Fig2 A&B

To gain a better understanding of how JG6 binds to cofilin, molecular docking was used to predict the potential binding sites and possible binding mode of JG6 to cofilin. A JG6 trimmer was selected as the docking probe. Our results indicated that JG6 binds to the actin-binding pocket of cofilin with high affinity. A close-up view of the binding interface was developed for an improved resolution (Fig. 3A). It was found that JG6 entered the actin-binding pocket of cofilin and docked on amino acids E42, K44, E50, K53 and D79 by forming hydrogen bonds.

Based on the simulation prediction, the impacts of these suggested amino acids on JG6 binding was examined. Surface plasmon resonance (SPR) assay was utilized to measure the binding affinity of JG6 to E42A, K44A, D79A, E42A/D79A and K44A/D79A cofilin mutants, which were expressed and purified in E. coli cells. It was revealed that only K44A/D79A among the 5 mutants significantly disrupted JG6 binding, resulting in nearly a 3600-fold decrease in binding affinity to JG6 (Fig. 3B and 3C). Meanwhile, none of the mutants affected the actin-binding and severing and depolymerization activities of cofilin (Fig. 3D).

For further confirmation, we examined whether introduction of K44A/D79A mutants would eliminate the impact of JG6 on actin polymerization. MTLn3 cells were transfected with cofilin siRNA to eliminate endogenous cofilin. Silent mutations were introduced to wildtype cofilin and K44A/D79A mutant to generate resistance to siRNA disruption. By measuring F-actin/G-actin ratio in cells transfected with wildtype cofilin and K44A/D79A mutant, we found that K44A/D79A mutant also completely eliminated JG6 modulated F-actin/G-actin ratio change (Fig. 3E).

All the data above support a mechanism that JG6 binds to cofilin, blocks the actin binding pocket, disrupts the interaction between cofilin and actin, and thereby hinders cofilin-dependent actin organization.

### JG6 suppresses cofilin-mediated cell migration

**Fig4:**
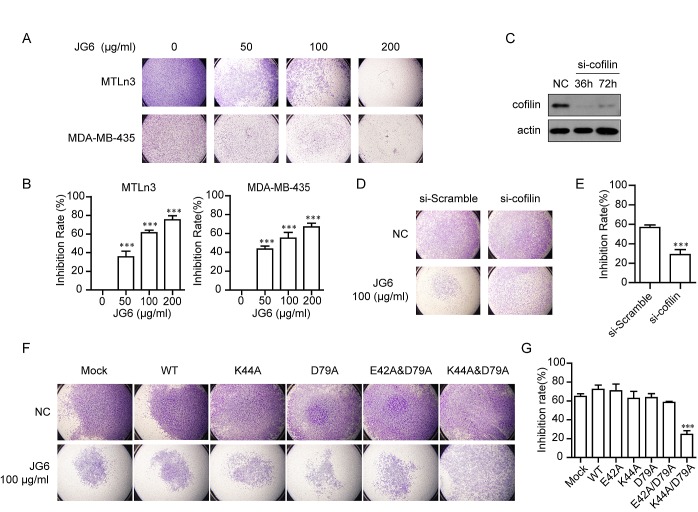
JG6 suppresses cofilin-mediated cell migration (A) JG6 inhibits serum-induced migration of breast cancer MTLn3 cells and MDA-MB-435 cells. The inhibitory effect of JG6 (50, 100 and 200 μg/ml) on cell migration was assessed using a Transwell migration assay as described in Materials and Methods. Representative pictures of three independent experiments with similar results were shown (magnification, 40×). (B) Cells that migrated into the lower chamber in (A) were counted. The data shown were the mean±S.E. of three independent experiments assuming no inhibition rate in the absence of JG6.(C) MTLn3 cells were transfected with scramble or cofilin siRNA for the indicated time, then subjected to Western blot analysis. (D&F) MTLn3 cells transfected with scramble siRNA or cofilin siRNA(D) and the mutant-cofilins or empty vector (Mock) (F)for 48 h. The inhibitory effect of JG6 (100 μg/ml) on migration was analyzed using Transwell migration assay, and representative images were shown. (E&G) The inhibition rate percentages of JG6 were determined, E for D and G for F, based on the cell numbers that migrated into the lower chamber. The mean±S.E. of three independent experiments are shown. ***, p < 0.001.

The defects in actin cytoskeleton organization are known to result in impaired migration of cancer cells. We investigated the impact of JG6 on the migration of breast cancer cells in vitro using a Transwell assay. As shown in Fig. 4A and 4B, in the absence of JG6, MDA-MB-435 or MTLn3 cells freely passed into the lower chamber. 12-hours treatment with JG6 at doses of 50, 100, and 200 μg/ml significantly and dose-dependently reduced the number of migrated cells. 200 μg/ml of JG6 yielded 75.3% (MTLn3) and 66.9% (MDA-MB-435) inhibition compared with the untreated control (*P* < 0.001). Meanwhile, JG6 treatment exhibited negligible cytotoxic effects on 6 different breast cancer cell lines (Fig. S1), suggesting that JG6 significantly inhibited migration of breast cancer cells without affecting cell viability.

To further determine whether JG6 suppressed cell migration was mediated by impaired function of cofilin, cofilin was knocked down using siRNA in MTLn3 cells, which yielded an 80% reduction in cofilin level (Fig. 4C). Effects of JG6 on cell migration were dramatically reduced in cofilin-depleted cells (Fig. 4D and 4E). Further, introduction of K44A/D79A mutant into cofilin-depleted cells remarkably rescued JG6 suppressed breast cancer cell migration (Fig. 4F and 4G). It was interesting to note that siRNA-medicated depletion or overexpression of cofilin barely affected cell mobility. We speculate that functional cofilin located at the dynamic protrusion represents only a very small proportion of cofilin pool in the cell. The alteration of its protein level, rather than disrupting its activity, may not be able to affect its functional output.

In addition to cell migration, cofilin activity is required for determining the direction of the protrusion in chemotaxis responding to chemotactic stimulation [12]. In a chemotaxis assay, control cells exhibited protrusion toward a gradient of EGF (the white asterisk indicated the position of the pipette) whereas treatment with JG6 eliminated protrusion toward the EGF source (Fig. S2). These data collectively suggested that JG6 suppressed cell migration was medicated by impaired function of cofilin.

### JG6 inhibits breast cancer metastasis

**Fig 5:**
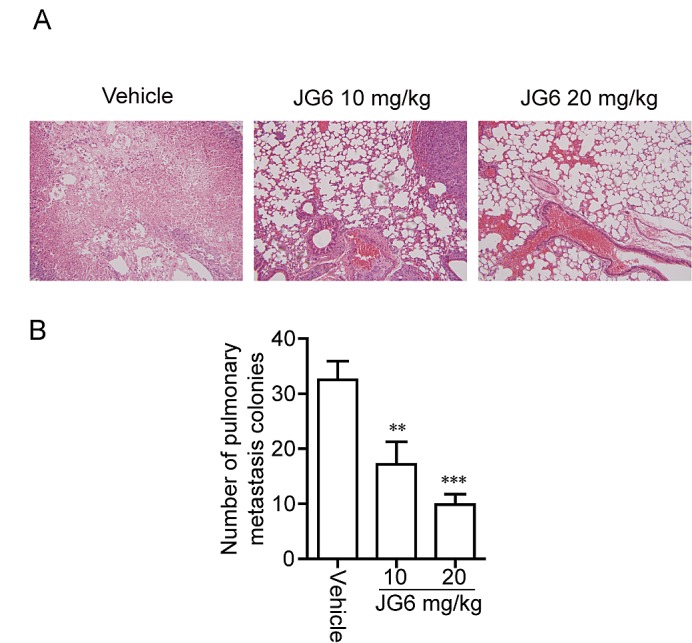
JG6 inhibits breast cancer metastasis (A) Effect of JG6 on lung metastasis of MDA-MB-435 breast carcinoma orthotopic xenografts in nude mice. Top, representative photograph of metastatic nodules on lungs with H&E staining (magnification, 200×). (B) The histogram shows the inhibitory action of JG6 on the number of pulmonary metastatic nodules. Columns, mean of a typical experiment; bars, SE.

Cell migration is critically required for the complex, multistep process of cancer metastasis [24, 25]. We finally intended to explore therapeutic chances of JG6 in overcoming metastasis of cancer. The anti-metastasis effect of JG6 was examined using a spontaneous metastasis assay. Hypodermic injection and inoculum of human breast cancer MDA-MB-435 cells into female athymic nude mice caused a significant increase in the number of pulmonary metastatic nodules. In contrast, daily subcutaneous (s.c.) administration of JG6 for 6 weeks (10 and 20 mg/kg) caused a dramatic and dose-dependent decrease in the number of pulmonary metastatic nodules, yielding inhibition rates of 46.9% and 68.8%, respectively (Fig. 5A and 5B). JG6-treated mice survived the whole study (except one died of operation mistake) and showed no signs of toxicity or body weight loss throughout the experiments (Table. S2). These results suggest the potential of JG6 in cancer therapy via hindering cancer metastasis.

## DISCUSSION

Dynamic cytoskeletal changes, as the foundation of cell structure, morphology and motility, are critical for cell-matrix interactions, focal contact disassembly, chemotaxis and invasion. To switch from a stationary state to a migratory state, the actin cytoskeleton has to be reorganized from F-actin in the form of stress fibers to G-actin monomers, and successively high order structures[20]. Assembly of actin filaments from their monomeric subunits can suffice to produce a protrusion, which is often the first step in cell locomotion. To disturb the actin dynamic will reduce cell motility[26].

Despite the apparently essential role in modulating cell mobility, the strategy to manipulate actin dynamics for cancer therapy appeared more complicated than expected. So far, only a few peptide or depsipeptide families have been reported to successfully inhibit actin depolymerization[27]. The most well-known class of compounds, including phalloidin and jasplakinolide, does so by binding to a well-characterized phalloidin-binding site on actin[28, 29]. In contrast to the diverse drugs targeting tubulin, therapeutic development of this class of compounds has been restricted due to the associated adverse effects of pulmonary edema and hemorrhage[30], which is recognized as a result of interference with the phalloidin-binding site on actin[31, 32]. Therefore, compounds circumventing direct actin-binding sites may increase the therapeutic opportunities by avoiding such adverse effects. In the present study, we have identified a marine-derived oligosaccharide JG6 that can effectively inhibit actin depolymerization, which was further translated into potent inhibitory effects on cell migration and tumor metastasis with little toxicity. Mechanistic studies have revealed that the actin-binding protein cofilin is the cellular target of JG6. JG6 blocks the interaction between cofilin and actin and hence disrupts cofilin modulated actin dynamics. Target validation of JG6 in vivo at the moment will be technically challenging. Molecular imaging of JG6 by conjugating to fluorescein could be an option. Our findings may highlight the strategy of targeting actin-binding proteins, such as cofilin or cofilin regulators such as LIMK1/2[33, 34], rather than actin itself for cancer therapy.

JG6 is structurally characterized as a 1, 4-linked β-D-mannurarate of pyranohexuronic acid residues, bearing an average of 1.5 sulfates per sugar residue at the 2-hydroxyl, partial 3-hydroxyl and 6-carboxyl groups, with an additional C1 carboxyl group at the reducing end. This structure endows JG6 appreciable negative charges, which may allow JG6 forming a high-affinity interaction with cofilin. Our results from molecular docking and mutagenesis studies identified the amino acids of cofilin critical for its interaction with JG6. Interestingly, these amino acids are located at the actin binding pocket of cofilin. These findings support a model that JG6 competes with actin in the interaction with cofilin. It is worthwhile to mention that although electrostatic charges initiate attachments between sugars and proteins, the structure-based conformation is more likely to dominate the ultimate outcomes. We have found that some other marine-derived oligosaccharides, with the same negative charges as JG6 but different sugar compositions, did not exhibit the same effect as JG6 (unpublished data). Though the structural components responsible for interactions between cofilin and sulfated oligosaccharides have not been well characterized, sugar backbone, distribution of sulfation as well as chain length seems important. With studies continuing to explore the structure-based mechanisms of action of JG6, it is hoped that these results will add impetus to the search for cofilin-targeted carbohydrate-based anti-cancer agents. Our study provides important insights into oligosaccharides as a new and hitherto unrecognized therapeutic class[36]. The unique affinity to proteins of this class of compounds may open new therapeutic opportunities, in particular for those targets challenging to access by small-molecule inhibitors.

Taken together, this study demonstrates for the first time that an oligosaccharide JG6 binds cofilin at the actin binding pocket and thereby inhibits actin turnover by restricting actin depolymerization. This mechanism allows JG6 anti-metastasis efficacy and highlights JG6 as a lead molecule in cancer therapy. In addition, our results identify cofilin as a key regulator in motility-driven cancer metastasis, which reinforces the view that cofilin may be a tractable and attractive target for cancer therapy.

## MATERIALS AND METHODS

### Materials

JG6 was obtained by semisynthesis following sulfate modification by reacting its precursor with ClSO_3_H in formamide. Briefly, oligomannurarate was added to sulfating reagents containing formamide and ClSO_3_H, and reacted for 3 hours. The pH of the products was adjusted to 7.0 with 4 mol/L NaOH and desalted with Sephadex G-10. The product peak was pooled and freezedried. The molecular weights of JG6 and its precursor were analyzed by high-performance gel permeation chromatography with a G3000PW×l column (300 mm×7.8 mm) (TOSOH, Japan). Cofilin, p-cofilin, Erizin, p-Erizin, p-VASP antibodies were from Cell Signaling Technology (Actin Reorganization Antibody Sampler Kit #9967); PLCγ1 (#2822) and p-PLCγ1 (#2821) antibodies were from Cell Signaling Technology. N-WASP (sc-20770), Arp2 (sc-15389), Arp3 (sc-15390), β-Actin(sc-130301), GAPDH (sc-166574) antibodies were from Santa Cruz Biotechnology: JG6 mAb was generated by hybridoma fusions of BALB/C mouse spleen cells and NS-1 myeloma cells. This mAb displays high affinity for JG6 with a KD value of 2.3×10^−9^ M, as determined using SPR. Analysis of the rate of cross-reactivity in an ELISA assay showed that the mAb did not react with the carrier proteins, BSA or OVA.

### Plasmids and Mutation

Vector pcDNA3.1-myc-His(A), pBAD-His(A) were obtained from Invitrogen. Cofilin mutants were generated using the Muta-direct Site-directed Mutagenesis Kit (Beijing SBS Genetech Co, Ltd, Beijing, China). Primers for generating cofilin siRNA-resistant mutations were: sense-GATGGTGTCATCAAAGTCTTTAACGACATGAAGGTGCG; antisense-CGCACCTTCATGTCGTTAAAGACTTTGATGACACCATC. Primers for generating cofilin mutants were: E42A sense- GCT CTTCTGCCTGAGTGCGGACAAGAAGAACATCA; E42A antisense- TGATGTTCTTCTTGTCCGCACTCAGGCAGAAGAGC; K44A sense- CTCTTCTGCCTGAGTGAGGACGCGAAGAACATCA; K44A antisense- TGATGTTCTTCGCGTCCTCACTCAGGCAGAAGAG; D79A sense- TGCTGCCAGATAAGGCCTGCCGCTATGCCC; D79A antisense- GGGCATAGCGGCAGGCCTTATCTGGCAGCA.

### RNA Interference

Shanghai GenePharm (Shanghai, China) synthesized short interfering RNA (siRNA) sequences specifically targeting cofilin: sense-AAGGUGUUCAAUGACAUGAAATT, antisense-UUUCAUGUCAUUGAACACCUUTT.

### Cell Culture

Human breast carcinoma cells MCF-7, MDA-MB-231, MDA-MB-468, MDA-MB-435 and BT-474 were obtained from ATCC and maintained in appropriate medium as suggested by ATCC. MTLn3 and GBA-MTLn3 cells (gifts of professor John S. Condeelis, Albert Einstein College of Medicine, Bronx, NY), rat mammary adenocarcinoma cell lines described by Chan et al.[35], were grown in α-MEM containing 5% FBS.

### FCM analysis

JG6-fluorescein-5-isothiocyanate (FITC) was used to detect the binding and entry of JG6 to cells. MDA-MB-435 and MTLn3 cells were plated into 6-well plates incubated at 37°C in a humidified incubator with 5% CO_2_ for 24 hours and then 100 μg/mL JG6-FITC was added. After incubated with JG6-FITC for the indicated time, cells were harvested and washed three times with PBS, then analyzed by FCM with a 488-nm laser excitation and a 513-nm emission filter. Data were analyzed with CellQuest software. For live cell fluorescence analysis, cells were seed into Nunc™ glass bottom dishes for 24 hours. Before and after the addition of JG6-FITC, cells were analyzed and photographed under a fluorescence microscope (OLYMPUS IX71).

### Transwell Assay

Migration of MDA-MB-435 and MTLn3 cells were evaluated using a Transwell assay as previously described[11]. Briefly, cells were seeded to the upper compartment of each well (1.5×10^4^ cells/well) in the presence or absence of JG6 with serum-free culture media. The lower compartment contained 600 μL of complete culture media supplemented with 10% FBS. After 12-hours incubation at 37°C, cells were fixed and stained with 0.1% crystal violet. The inhibition rate was calculated as:[1-(JG6 treated group/control group)] × 100%.

### Molecular modeling

DOCK 4.0 was employed for conformational screening, based on the X-ray crystal structure of the cofilin protein reported in the Brookhaven Protein Database. Residues within 5Å of the active center, actin binding domain of cofilin was showed as the binding pocket for docking. During docking simulation, different conformational isomers of tri-mannurarate were used to present JG6.

### Protein purification

E. coli BL21 transformants harboring the expression vectors pBAD-His-cofilin or cofilin mutation were cultured in RM media (1× M9 salts-2% Casamino acids-1 mM MgCl2) plus 0.2% glucose with 150 μg/mL ampicillin at 37 °C in a buffered flask with constant shaking at 300 rpm, until the cell cultures reached an OD600 of 0.8. L-(+)-arabinose was added to culture to a final concentration 0.2% (w/v), and the cell cultures were then allowed to grow for an additional 4 h at 30 °C. Cells were pelleted by centrifugation at 3,000 × g for 15 min, washed once in half of the original volume with 10 mM Tris-HCl buffer, pH 8.0, containing 0.1 M NaCl. Cell pellets were resuspended in 1:20 of the original volume in extraction buffer containing 50 mM K_3_PO_4_, pH 8.0, 10 μM PLP, 0.1 M NaCl, 1 mM PMSF, 2 mM EDTA, and protease inhibitor. The cells were lysed by sonication. The precipitate was removed by centrifugation. The supernatant was applied to Ni-NTA agarose column. After the column was washed with washing buffer (20 mM Na_3_PO_4_, 500 mM NaCl, pH 6.0) three times, the His-cofilin proteins were eluted with elution buffers that had increasing imidazole concentrations at 500 mM.

### SPR assay

SPR biosensor measurements (Bio-Rad ProteOn XPR36) were used to evaluate the interactions between JG6 and cofilin or cofilin mutants. In this assay, 10 mM Tris-HCl buffer (pH 8.0) containing cofilin or cofilin mutants at concentrations of 0.312, 0.625, 1.25, 2.5, or 5 μM were passed over the JG6 sensor chip surface for 2 minutes at a flow rate of 5 μl/min. Changes in mass were measured, and the sensorgrams were recorded in real time and analyzed after subtracting the control.

### Preparation of F- and G-actin Extracts

The concentration of F-actin and G-actin in cells was obtained using an F-actin/G-actin assay kit (catalog number: BK 037, Cytoskeleton, Denver, CO). Briefly, cells were rinsed with phosphate-buffered saline at 25 °C and scraped and homogenized in a lysis and F-actin stabilization buffer (LAS1). F-actin was then separated from G-actin by centrifugation at 100,000 × g for 60 min at 37 °C. The F-actin-containing pellet was resuspended in ddH_2_O containing 2 μm cytochalasin D at a volume equivalent to the G-actin-containing supernatant volume. The resuspended F-actin pellet was kept on ice for 60 min with mixing by pipette every 15 min to dissociate F-actin. After dissociation, dissociated F-actin was centrifuged at 14,000 g for 10 min at 4 °C. The F-actin and G-actin preparations were then assayed for protein.

### F-actin depolymerizing/severing activity assay

Mg^2+^-bound actin (4 μM; 5% pyrene-labelled) was polymerized for 12 h at 20 °C. Depolymerization was initiated by a 12-fold dilution in 10 mM Hepes/KOH/16 mM Tris/HCl/2 mM MgCl_2_/100 mM KCl/0.5 mM EGTA/0.1 mM dithiothreitol (pH 7.2) containing 2 μM WT or mutant cofilin. The decline in fluorescence was monitored immediately. JG6 was added when necessary, depending on certain application.

### MTT cytotoxicity assay

Human breast cancer cells were seeded into 96-well plates, attached overnight and subsequently exposed to different concentrations of JG6 for 48 h. Cytotoxicity to breast cancer cells were assessed by MTT assay as previously described[11]. The cytotoxicity was measured as inhibition rate which was calculated as:[1-(A570 treated/A570 control)] × 100%.

### Co-immunoprecipitation (co-IP)

For total protein extraction, the indicated cell lines were lysed in buffer consisting of 25 mM HEPES (pH 7.5), 150 mM NaCl, 10 mM MgCl_2_, 0.1 mM EDTA, 1 mM sodium orthovanadate, 1% Nonidet P-40, and supplemented with a protease inhibitor cocktail (Roche, Indianapolis, IN) and then centrifuged at 12,000 g at 4°C for 15 minutes. For co-IP, the protein lysates (1 mg) were incubated with the JG6 antibodies with rocking for 4 hours at 4°C, followed by the incubation of protein A/G plus Agarose (Santa Cruz) for 6 hours to overnight at 4°C with rocking. The bound proteins were then eluted using 0.2% sodium dodecyl sulfate.

### Immunoblot Analysis

Both total protein lysates and eluted proteins from the co-IP experiment were separated by sodium dodecyl sulfate polyacrylamide gel electrophoresis and transferred to Hybond C Extra nitrocellulose membranes (GE Healthcare, Chalfont, St.Giles, UK). Membranes were incubated in blocking solution containing 5% nonfat dry milk for 1 hour at room temperature. The membranes were incubated at 4°C overnight with the appropriate primary antibodies immunoblotting for GAPDH or β-actin served as a protein loading control. All experiments were performed at least three independent times.

### Spontaneous Metastasis Assay and Tumor Growth Inhibition

Female athymic nude mice, ages 4 to 5 weeks, were anesthetized with chloral hydrate, Hypodermic injection an inoculum of human breast cancer MDA-MB-435 cells (5×10^7^) through a 27-gauge needle. The well-developed tumors were cut into 1-mm^3^ fragments and transplanted s.c. into the right flank of nude mice using a trocar. When the tumor reached a volume of 100 to 200 mm^3^, mice were divided into three experimental groups, specifically (a) untreated (n = 12); (b) JG6 10 mg/kg (n = 6); and (c) JG6 20 mg/kg (n = 6). JG6 was s.c. administrated through the tail vein once a day for 6 weeks thereafter. Tumors and body weight of mice were measured individually twice per week. Mice were sacrificed 5 days later after the final therapy. The lungs were removed, some were fixed with Bouin's solution, and metastatic nodules were counted.

### Statistics

Student t-test and analysis of variance (ANOVA) were performed using Statview. P < 0.05 was considered significant and P < 0.01 as highly significant. Five parallel samples were prepared in each group and all experiments were replicated at least three times.

## SUPPLEMENTARY MATERIALS TABLES AND FIGURES






