# Supplementary material for: JG6, a novel marine-derived oligosaccharide, suppresses breast cancer metastasis via binding to cofilin
Source: Oncotarget. 2014 May 12;5(11):3568–78. doi: 10.18632/oncotarget.1959 (PMC4116503; doi:10.18632/oncotarget.1959)
Supplement: Supplementary file 2 [file oncotarget-05-3568-s002.pdf]

Supplemental Table 2: Effect of JG6 on MDA-MB-435 spontaneous metastasis assays in mice

Groups	Dosage & Administration route		Animal numbers		Body weight (g)		Average lung weight (g)	Animal number(s) in this range of pulmonary metastasis colonies				Number of pulmonary metastasis colonies (mean±SD)	IR(%)
			start	end	start	end		0	1-15	16-30	31-45		
Vehicle	0.4ml/mouse, qd×6w	s.c	12	12	19.9	22.9	0.45±0.21	0	0	6	6	32±12	
JG6	10mg/kg, qd×6w	s.c	6	5	20.7	23.6	0.32±0.08	0	2	3	0	17±9**	46.9
JG6	20mg/kg, qd×6w	s.c	6	6	20.8	22.6	0.31±0.08	0	5	1	0	10±5***	68.8

t student's test vs vehicle，p<0.01，***p<0.001**
