# Supplementary material for: JG6, a novel marine-derived oligosaccharide, suppresses breast cancer metastasis via binding to cofilin
Source: Oncotarget. 2014 May 12;5(11):3568–78. doi: 10.18632/oncotarget.1959 (PMC4116503; doi:10.18632/oncotarget.1959)
Supplement: Supplementary file 3 [file oncotarget-05-3568-s003.pdf]

JG6, a novel marine-derived oligosaccharide, suppresses breast cancer metastasis via binding to cofilin

Supplementary Material

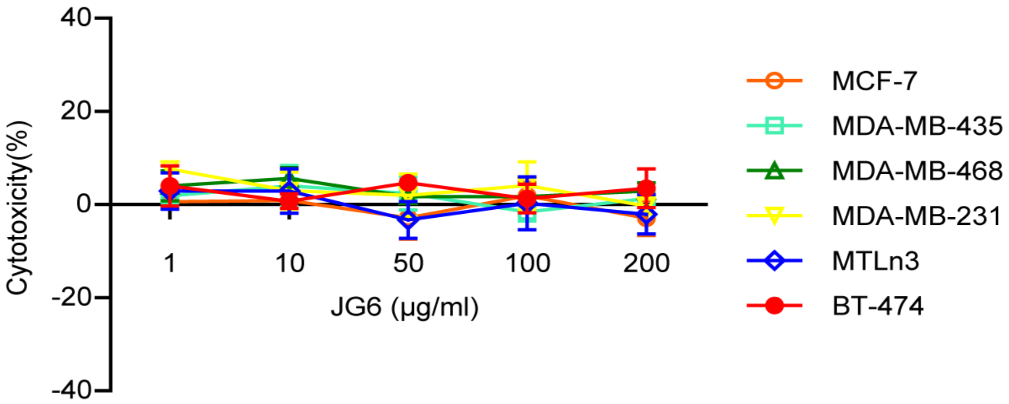

Fig S1: JG6 does not affect cell viability. Cells were seeds in 96-well plates, cell viability was determined after 24 h by MTT assay. Data were the mean± S.D. (n = 3).

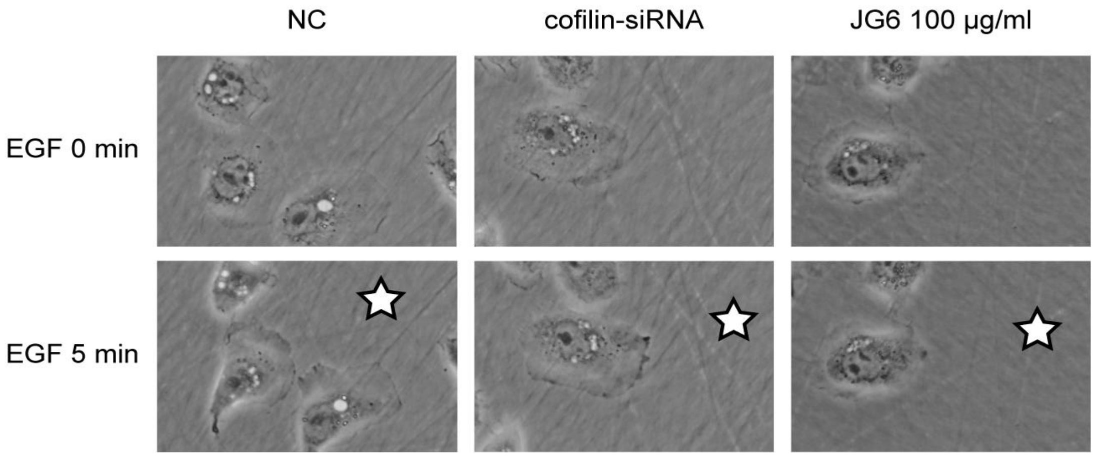

Fig S2: JG6 inhibits protrusion formation in chemotaxis. Still images at 0 and 5min of control and cofilin KD MTLn3 cells stimulated with an EGF-filled micropipette at various time points after stimulation (the white asterisk indicates the approximate position of the pipette tip; arrows indicate areas of protrusion).
